# Submicroscopic *Plasmodium falciparum* malaria and low birth weight in an area of unstable malaria transmission in Central Sudan

**DOI:** 10.1186/1475-2875-12-172

**Published:** 2013-05-28

**Authors:** Amal H Mohammed, Magdi M Salih, Elhassan M Elhassan, Ahmed A Mohmmed, Salah E Elzaki, Badria B El-Sayed, Ishag Adam

**Affiliations:** 1 Faculty of Medicine, University of Khartoum, P. O. Box 102, Khartoum, Sudan; 2 Faculty of Medical Laboratory Sciences, University of Khartoum, Khartoum, Sudan; 3 Faculty of Medicine, University of Gezira, P.O. Box 816, Medani, Sudan; 4 Faculty of Medicine, The National Ribat University, P.O. Box 1157, Khartoum, Sudan; 5 Department of Epidemiology, Tropical Medicine Research Institute, National Centre for Research, Khartoum, Sudan

**Keywords:** *Plasmodium falciparum*, Submicroscopic infection, Pregnancy, Placenta, Parasite, Low birth weight

## Abstract

**Background:**

Malaria, which frequently occurs in pregnant women in the tropics, is a leading cause of maternal anaemia and low birth weight (LBW) in infants. Few data exist concerning malaria infections that are present at submicroscopic levels during pregnancy and their LBW delivery in babies.

**Methods:**

A case–control study (87 in each group) was conducted at the Medani Hospital, Central Sudan. Cases were women who had LBW deliveries where the infants weighed < 2,500 g. Controls were parturient women without having LBW babies. Obstetrical and medical characteristics were gathered from both groups through structured questionnaires. Both cases and controls were investigated for malaria using microscopic blood film analysis, placental histology and polymerase chain reaction (PCR). Microscopic and PCR analyses were conducted on maternal peripheral blood, placenta, and umbilical cord samples. Infant weights were recorded immediately after birth.

**Results:**

*Plasmodium falciparum*-positive blood films were not obtained from any of the women (cases or controls). Twenty-seven (31.0%) *versus* 22 (25.3%) (P = 0.500) of the cases and controls, respectively, had placental malaria infections as determined by histological examination. In comparison to the controls, the submicroscopic malaria infection prevalence rates were significantly higher in the cases; 24 (27.6%) *vs* six (7.0%), P < 0.001. Multivariate analysis showed that while malaria infection of the placenta (based on histology) was not associated with LBW, submicroscopic *P. falciparum* infection (OR = 6.89, 95% CI = 2.2–20.8; P = 0.001), or a combination of histologically determined and submicroscopic infections (OR = 2.45, 95% CI = 1.2–4.9; P = 0.012), were significantly associated with LBW.

**Conclusion:**

In Central Sudan, pregnant women were at a higher risk of having an LBW delivery if they had submicroscopic infections rather than a histological diagnosis of placental malaria.

## Background

Malaria is a big public health problem in tropical countries, especially sub-Saharan Africa. Around 125 million pregnant women live in malaria-endemic areas and 32 million of these are at risk of malaria in sub-Saharan Africa [1,2]. Malaria during pregnancy can lead to maternal anaemia and low birth weight (LBW) delivery, the latter of which is the main risk for neonatal and infant morbidity and mortality [3-5]. Malaria during pregnancy is caused by parasites sequestering in the placenta where selection of pregnancy-associated *Plasmodium falciparum* erythrocyte membrane protein-1 (*Pf*EMP-1) variant surface antigen occurs [6]. Thus, placental malaria infection (especially in areas of unstable malaria transmission) may be detected in the absence of peripheral blood parasitaemia [7,8]. Sequestration of malaria parasites in the placenta may lead to functional damage of placental villi, disturb the foetomaternal compartment and lead to LBW [9,10].

Although placental histology is the ‘gold standard’ for malaria diagnosis during pregnancy, it is often not available in most settings where malaria is endemic such as sub-Saharan Africa [11]. Polymerase chain reaction (PCR) is an alternative diagnostic tool that is widely used to diagnose malaria infection during pregnancy [12,13]. However, there are few published studies, as well as inconsistent findings on associations between submicroscopic malaria infections and LBW [14-16].

In Sudan, malaria during pregnancy is a big health problem because women are more susceptible to malaria (peripheral, placental and submicroscopic infections) during pregnancy regardless of their age or parity and severe cases of malaria have been observed [7,12,17-19]. Malaria has many adverse effects on pregnancy and its outcome and it is a leading cause of maternal and perinatal mortality in Sudan [20-22].

The aim of this study was to build upon the previous work on placental malaria and LBW in Sudan [23-25]; specifically, to investigate the effect of submicroscopic levels of malaria parasites during pregnancy on birth weight. The study took place in the Medani Maternity Hospital in Central Sudan.

## Methods

A case–control study was conducted during the post rainy season (September to November) 2010 at the labour ward of the Medani Maternity Hospital, Central Sudan. Central Sudan is characterized by unstable malaria transmission and *P. falciparum* is the sole malaria parasite species in the area and the transmission during the rainy (July –September) and post-rainy season [26]. Medani Maternity Hospital is a tertiary hospital for women who receive antenatal care at the hospital or are referred from other clinics and hospitals, and women who live close to the hospital facility. Women with high-risk pregnancies are referred to the hospital. However, the referral criteria are not strictly adhered to and many women without a high-risk pregnancy deliver at the hospital.

A total sample size was calculated to provide 80% power to detect the difference of 5% at α = 0.05 and assumed 10% of women would not respond or have incomplete data. In this study, a case represents a woman who had an LBW delivery (<2,500 g). A consecutive woman who delivered next to the case was taken as control for each case. Controls were parturient women who have no LBW delivery (≥ 2,500 g). Women pregnant with twins and those with hypertension, diabetes mellitus or antepartum haemorrhage were excluded from the study in both case or controls groups. After obtaining a signed informed consent, women in the case and control groups were enlisted to participate in the study. Information on socio-demographics, obstetrics history, medical characteristics and antennal attendance were gathered through structured pretested questionnaires. Women in both groups were asked if they used bed nets and if they had experienced malaria infections in the index pregnancy. Body mass index was calculated by measuring maternal weight and height, which was expressed as weight (kg)/height (m)^2^. Babies were weighed immediately following birth to the nearest 10 g on a Salter scale. Scales were checked for accuracy on a weekly basis. The gender of each baby was recorded.

### Giemsa-stained blood smears and light microscopy

Peripheral blood films were prepared from the mother, along with placental and umbilical cord samples. Both thick and thin blood films were prepared and stained with10% Giemsa and the parasite counts were obtained by counting the number of asexual parasites per 200 leukocytes assuming a leukocyte count of 8,000 leukocytes/μl (for thick films) or per 1,000 red blood cells (for thin films); blood films were considered negative if no parasites were detected in 100 oil immersion fields of a thick blood film.

### Placental histology

Full thickness placental blocks around 3 cm were taken from the placenta and kept in neutral buffered formalin for histopathological examination. Buffer was used to prevent formalin pigment formation, which has similar optical characteristics and polarized light activity as malaria pigment [27]. Placental malaria infections were characterized as previously described by Bulmer et al. [28]: uninfected (no parasites or pigment), acute (parasites in intervillous spaces), chronic (parasites in maternal erythrocytes and pigment in fibrin, or cells within fibrin and/or chorionic villous syncytiotrophoblast or stroma), and past (no parasites and pigment confined to fibrin or cells within fibrin). The slide was read by a pathologist (AAM) who remained blind about the clinical characteristics and the arms of the study.

### Parasite DNA extraction and PCR

*Plasmodium falciparum* DNA extraction and PCR assays were performed as described in the recent work [12,24]. In brief, three drops of blood were collected onto a piece of filter paper from maternal peripheral blood, the maternal side of the placenta, and the umbilical cord. These samples were air-dried and stored at ambient temperature in individual sterile plastic bags. The specimens were transported for processing and analysis in the lab in Khartoum. Approximately 25 μl (corresponding to approximately one third of a spot) of blood was punched out from the dried blood spots. The filter paper piece was washed with distilled water and placed directly in a PCR reaction tube containing 25 μl of all the PCR reaction components. A negative control sample with no template DNA and an internal positive control were used for quality control purposes. Genomic DNA was checked, in an assay based on a nested PCR, for DNA from *P. falciparum*[29]. PCR assays were performed by two of the team (HMI and MIE) who were both blinded to the clinical and the histology study data.

### Definitions

The malarial infection status of a participant was defined as any sample positive by microscopic and submicroscopic analysis or placental histology. Microscopic *P. falciparum* infections where determined at delivery from peripheral blood, placenta, and umbilical cord blood smears. Submicroscopic infections were defined as those participants with negative thick blood smears, but who were positive for *P. falciparum* based on the PCR results for peripheral blood, placenta, and umbilical cord samples. The *P. falciparum* negative group was defined by the absence of *P. falciparum* in thick blood smears, placental histology and PCR of peripheral blood, placental and umbilical cord samples.

### Ethics

The study received ethical clearance from the Research Board at the Faculty of Medicine, University of Khartoum, Sudan.

### Statistics

Data were analysed using SPSS for Windows version 16.0. Data means and proportions were compared by Student’s-test, *X*^2^ and Fisher’s exact tests as appropriate. Univariate and multivariate analyses were performed using LBW as the dependent variable. Maternal socio-demographic characteristics (age, parity, education, residence, antenatal care), malaria infection status (diagnosed by histology), and submicroscopic blood analysis (maternal peripheral blood, placenta and cord, or a combination) were included as possible influencing factors. P < 0.05 was regarded as significant.

## Results

Out of 820 deliveries, 98 (12.0%) were of LBW babies. Eighty-seven of these women with LBW deliveries fulfilled the inclusion criteria and had complete data, including placental histology and PCR diagnoses, and were, therefore, included in the final analyses. Such data were compared with an equal number of controls with complete data. There were no significant differences between the two groups (case or control) in their level of education, residence, antenatal care attendance and other socio-demographic characteristics (Table 1). While the mean (SD) maternal age was significantly lower [25.5 (5.7) *versus* 27.6 (6.4) years; P = 0.022], there was no significant difference in the body mass index [23.8 (3.3) *vs* 24.3 (2.2) kg/m^2^] in the LBW delivery *vs* the control women, respectively. The bed net coverage was low in both groups, but there was no statistically significant difference (Table 1).

**Table 1:** Socio-demographic characteristics and malaria status in the cases and controls

***Number (%) of***	**Low birth weight (N = 87)**	**Controls (*N*** **= 87)**	***P***
Primigravidae	31(34.3)	22(18.9)	0.186
Antenatal care ≤ thee time	62(14.7)	68(23.1)	0.446
Educational level < secondary	36(25.2)	38(16.8)	0.151
Rural residency	61(70.1)	57(65.5)	0.627
Bed net coverage	15(14.7)	12 (16.1)	0.675
Male gender	47(54.0)	45(50.6)	0.760
Anaemia (haemoglobin < 11 g/dl)	74(85.1)	70(80.1)	0.246

### Malaria infections

**Figure 1:**
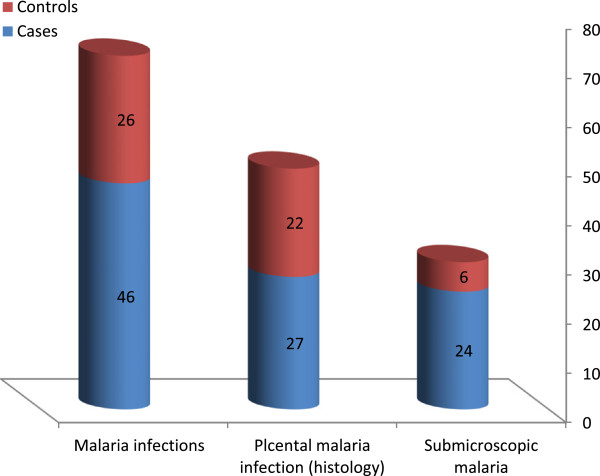
Malaria infection prevalence in the cases and controls.

No *P. falciparum*-positive blood films were obtained from maternal peripheral blood, placenta or cord samples in either the cases or controls. Twenty-seven (31.0%) *vs* 22 (25.3%) (P = 0.500) of the cases *vs* controls had placental malaria infections on histological examination. Three (3.4%), one (1.1%) and 23 (26.4%) *vs* two (2.3%), two (2.3%) and 18 (20.7%) of the placentae showed acute, chronic and past infection on histopathology examination in the two groups (case–control), respectively, while 60 (69.0.4%) *vs* 65 (74.7%) of them showed no signs of infection; P = 0.500, (Figure 1 and Table 2).

**Table 2:** Malaria status in the cases and controls

***Number (%) of***	**Low birth weight (N = 87)**	**Controls (*N*** **= 87)**	***P***
**All malaria infection (histology/submicroscopic**	46(53.0)	26(30.0)	**0.002**
**Placental malaria (histology)**	27(31.0)	22(25.3)	**0.500**
Acute	3(3.4)	2(2.3)	0.999
Chronic	1(1.1)	2(2.3)	0.999
Past	23(26.4)	18(20.7)	0.474
**Submicroscopic malaria**	24(27.6)	6(7.0)	**<0.001**
Maternal	0(0)	4(4.6)	0.129
Placental	19 (21.8)	2(2.3)	<0.001
Cord	6 (7.0)	0(0)	0.037
Maternal, placental and cord	1(1.1)	0(0)	0.999

In comparison to the controls, the prevalence of submicroscopic malaria infection was highly significant in the cases; 24 (27.6%) *vs* six (7.0%); P < 0.001. None (0%), 19 (21.8%) and six (7.0%) *vs* four (4.6%), two (2.3%) and none (0%) were maternal, placental or cord submicroscopic malaria infections in the two study groups, respectively. One case had a placental and umbilical cord submicroscopic malaria infection, Table 2.

Significantly higher numbers of the cases than the controls had malaria infections (placental malaria infections on histology/submicroscopic malaria infection); 46 (53.0%) *vs* 26 (30.0%), P = 0.002. Out of these malaria infections, six (five and one in the cases and controls, respectively) had histologically positive placental malaria infections as well as submicroscopic malaria infections (Figure 1).

### Effects of malaria infection on birth weight

**Figure 2:**
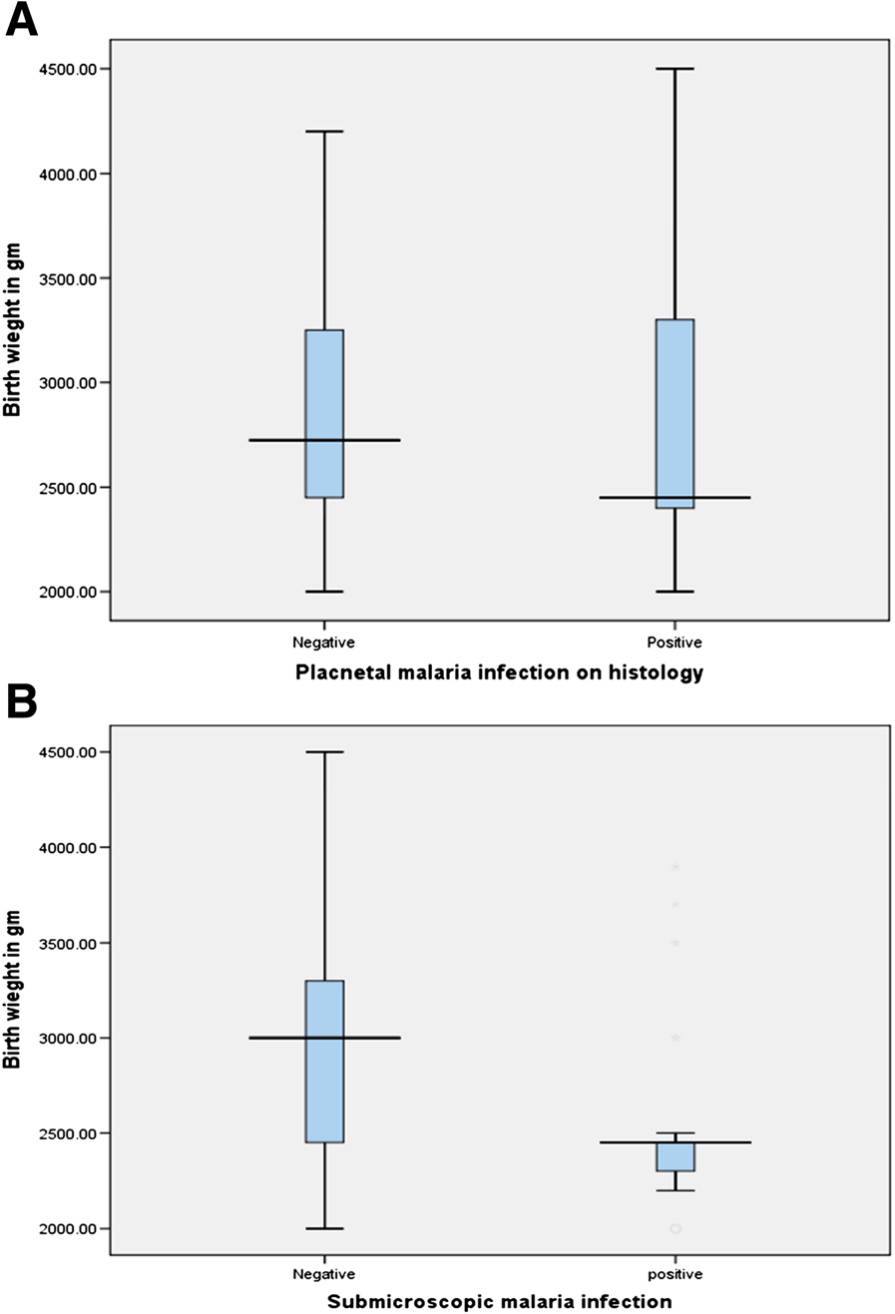
**Birth weight in placental (histology) and submicroscopic malaria.** (**A**) Birth weights in placental (histology) and (**B**) submicroscopic malaria.

The mean (SD) of birth weight was 2,387.2 (152) *vs* 3,319.2 (358) g, P < 0.001 in the cases and controls, respectively. While there was no significant difference in the mean (SD) of the birth weight between those who had placental malaria infections based on histology (in both groups, N = 49) and those who did not [2843.0 (601.0) *vs* 2834.7 (533.0) g], the mean (SD) of the birth weights was significantly lower in those who had submicroscopic malaria infection (in both groups, N = 30) than in those who had no evidence of a submicroscopic malaria infection [2551.7 (497.0) *vs* 2896.9 (545.0) g] (Figure 2).

### Malaria infection as a risk factor for low birth weight infants

In this multivariate analysis, while the presence of placental *P. falciparum* infection (by histology) was not associated with LBW, submicroscopic infections with this parasite (OR = 6.89, 95% CI = 2.2–20.8; P = 0.001) and all *P. falciparum* infections (histological or submicroscopic) were significantly associated with LBW (OR = 2.45, 95% CI = 1.2–4.9; P = 0.012), (Table 3).

**Table 3:** Factors associated with low birth weight in Medani Maternity Hospital, Central Sudan based on univariate or multivariate analysis

	**Univariate analysis**			**Multivariate analysis**		
**Variable**	**OR**	**95% CI**	***P***	**OR**	**95% CI**	***P***
Age	0.94	0.8–0.9	0.024	0.96	0.8–1.0	0.412
Primigravidae	1.64	0.8–3.1	0.139	0.93	0.6–1.3	0.706
Residence	0.81	0.4–1.5	0.517	0.50	0.2–1.1	0.104
Educational level < secondary	1.79	0.7–4.1	0.169	2.41	0.7–7.5	0.130
Lack of antenatal care	1.47	0.7–2.7	0.217	1.17	0.4–2.9	0.723
Body mass index	1.06	0.9–1.1	0.240	1.09	0.9–1.2	0.185
Haemoglobin	1.28	0.9–1.7	0.084	1.31	0.9–1.8	0.145
Male gender	0.88	0.4–1.6	0.697	1.08	0.5–2.1	0.821
Placental malaria infections (histology)	1.33	0.6–2.5	0.400	1.00	0.4–2.1	0.990
Submicroscopic malaria infections	5.14	1.9–13.3	0.001	6.89	2.2–20.8	0.001
All malaria infections	2.63	1.4–4.9	0.002	2.45	1.2–4.9	0.012

## Discussion

The main findings of the current study were as follows. While there was no significant difference in the prevalence of placental malaria by histological examination (31.0% *vs* 25.3; P = 0.500) between the two groups, significantly higher numbers of the cases had submicroscopic malaria infections than the controls (27.6% *vs* 7.0%; P < 0.001). While placental malaria infections that were positive by histology were not associated with LBW, submicroscopic malaria infections were, and this was a statistically significant finding. In fact, the impetus for this study arose out of the knowledge that two cross-sectional studies in Eastern Sudan failed to show significant associations between LBW delivery and placental malaria infection, as diagnosed by histology [7,18]. The prevalence (28.0%) of histologically determined placental malaria infections in both groups (cases and controls) in the current study is similar to the prevalence of the placental malaria infections recently observed in Eastern and Central Sudan [7,18,23].

Interestingly, in the current study only six of the malaria infections (28.0%) had both placental malaria infections (histology) and submicroscopic malaria infections. The performance of PCR *versus* histology for diagnosing placental malaria infections was recently investigated in the same hospital (Medani Hospital, Central Sudan) [24]. The low sensitivity and specificity was explained by the different nature of the infections detected by the different methods (histology and PCR) [24].

In the current study, while women with placental malaria infections (confirmed by histology) were not at risk of having an LBW delivery, women who had submicroscopic malaria infections had a seven-fold higher risk of such an event. In addition, birth weights were significantly lower in women who had submicroscopic malaria infections. Interestingly, it has been shown that pregnant women in Burkina Faso with submicroscopic malaria infections delivered infants that weighed significantly less than those delivered by women with no placental infections. Yet in the same study, delivery of LBW babies was not more common among women with submicroscopic placental malaria parasitaemia than those women without malaria [14]. In Gabon, it has been found that women with submicroscopic *P. falciparum* infections had a 13-fold higher risk of LBW delivery compared with non-infected pregnant women [15]. Remarkably, malaria infections in pregnant Kenyan women as estimated by real-time quantitative PCR were strongly associated with LBW delivery, but malaria detected by nested PCR showed a weaker association [30].

Conversely, the current study identified an association between submicroscopic malaria and LBW; this contrasts with previous reports from Malawi [16,31] and Ghana [32], where no statistically significant association between submicroscopic *P. falciparum* infection and LBW was observed.

One of the limitations of the current study was the inability, by its design, to investigate the other effects of submicroscopic *P. falciparum* malaria on pregnancy outcomes such as anaemia. This is because it was designed to investigate LBW through a case–control study; another study is needed if the effects of submicroscopic *P. falciparum* malaria on haemoglobin are to be investigated. The second limitation is the sample size, where the current study failed to yield enough maternal, placental and umbilical cord submicroscopic *P. falciparum* malaria infections to enable parasite genotyping to be conducted. Therefore, a larger cross-sectional study is needed to address the effect on haemoglobin and parasite genotyping.

## Conclusion

In Central Sudan, pregnant women were at higher risk of having an LBW delivery if they had submicroscopic infections rather than a diagnosis of placental malaria based on histology.

## Competing interests

The authors declare that they have no competing interests.

## Authors’ contributions

AHM and IA coordinated and carried out the study, and participated in the statistical analysis and procedures. EME and BBE participated in the clinical work and statistical analysis. MMS, AAM and SEE conducted the laboratory work. All the authors have read and approved the final version of this manuscript.
